# Remimazolam combined with propofol for intraoperative wake-up testing in scoliosis correction surgery: a prospective, blind, randomized controlled trial

**DOI:** 10.3389/fphar.2025.1685683

**Published:** 2025-11-24

**Authors:** Jing Luo, Da-Lin Chen, Zong-Long Lv, Hui Zhong, Yong-Hui Zhao, Yu-Jin Li, Xin-Nan Li, Jing-Ce Chen, Wen-Bao Shi, Yun Bo, Rong-Qian Lin, You-Zhi Yang, Sheng Lu, Wen-Sen Li, Hua Jin

**Affiliations:** 1 Faculty of Life science and Technology, Kunming University of Science and Technology, Kunming, China; 2 Department of Anesthesiology, The First People’s Hospital of Yunnan Province &The Affiliated Hospital of Kunming University of Science and Technology, Kunming, China; 3 Department of Anesthesiology, Simao District People’s Hospital, Pu’er, China; 4 Department of Orthopedics, The First People’s Hospital of Yunnan Province &The Affiliated Hospital of Kunming University of Science and Technology, Kunming, China

**Keywords:** scoliosis surgery, wake-up test, remimazolam, propofol, dexmedetomidine, neurophysiological monitoring

## Abstract

**Purpose:**

Remimazolam is a fast-acting benzodiazepine used for anesthesia and sedation, notable for its quick onset and reversible effects. Its role in wake-up testing during spinal deformity surgery is uncertain. This study assessed the effectiveness and safety of combining remimazolam with propofol during wake-up tests in scoliosis correction surgery.

**Methods:**

In this randomized trial, 54 scoliosis surgery patients were assigned to receive either propofol alone (Group P), propofol with dexmedetomidine (Group D), or propofol with remimazolam (Group R). Primary outcomes were wake-up time and quality; secondary outcomes included spinal monitoring, cerebral indices, hemodynamic stability, surgery duration, Visual Analog Scale and Behavioral Comfort Scale scores, satisfaction, and postoperative adverse events.

**Results:**

Mean wake-up times varied significantly across groups: 10.58 ± 1.60 min (Group P), 16.14 ± 2.64 min (Group D), and 6.69 ± 3.85 min (Group R) (*p* = 0.001). Post-hoc analysis revealed Group R had significantly shorter wake-up times than Group P (mean difference, 3.9 min; *p* = 0.0005) and Group D (mean difference, 9.4 min; *p* = 0.0001). Excellent wake-up quality was reported in 50% of Group P, 100% of Group D, and 94.4% of Group R (*p* = 0.0002). Group R also had a shorter mean operative duration (256.3 ± 74.4 min) compared to Group P (264.4 ± 60.4 min) and Group D (310.9 ± 57.9 min) (*p* = 0.03). Additionally, Group R showed significantly higher amnesia indices at multiple time points compared to Groups P and D (*p* < 0.05). Hemodynamic assessments revealed that Group D had significantly lower heart rates at T2, T3, and T4 than Groups P and R (*p* < 0.05). Group R also had a higher mean arterial pressure than Group P at T2 (*p* < 0.05). Surgeon satisfaction scores were significantly higher in Group R than in Groups P and D (*p* = 0.0001), and patient satisfaction scores in Group R were higher than in Group P (*p* = 0.0001). No significant differences were observed in other secondary outcomes (*p* > 0.05).

**Conclusion:**

The remimazolam-propofol combo for intraoperative wake-up testing in scoliosis correction surgery shows superior efficacy with quicker wake-up times, better wakefulness quality, and higher satisfaction, without affecting spinal neurophysiological monitoring. This suggests it as a promising sedative option for spinal deformity surgeries.

**Clinical Trial Registration:**

https://www.chictr.org.cn/bin/project/edit?pid=203900 as ChiCTR2300076802.

## 1 Introduction

Scoliosis is a complex spinal deformity influenced by genetic factors, bone growth, hormones, muscles, nerves, and environmental conditions. It primarily results in structural spinal alterations, musculoskeletal imbalances, and neural regulatory disturbances (Lacroix et al., 2023). If left untreated, scoliosis may cause psychological distress, chronic pain, limited mobility, decreased cardiopulmonary function, and impaired growth, significantly diminishing patients’ quality of life (Kontodimopoulos et al., 2018; Peng et al., 2020). Scoliosis can be categorized as idiopathic, congenital, or degenerative, with idiopathic scoliosis being the most common, particularly affecting adolescents aged 10–18 years. Idiopathic scoliosis exhibits higher prevalence in females and is associated with genetic predisposition, rapid growth periods, and neuromuscular imbalances (White and Panjabi, 1976; de Seze and Cugy, 2012; Peng et al., 2020). For patients presenting with Cobb angles ≥45°, spinal correction surgery is the preferred treatment, as it enhances physical appearance, overall health, and quality of life, while preventing neurological deterioration and deformity progression (Schulze et al., 2015; Putzier et al., 2016; Rahyussalim et al., 2016).

However, scoliosis correction surgery (SOSCS) is a complex procedure with an inherent risk of iatrogenic spinal cord injury, potentially leading to severe consequences (Rahyussalim et al., 2016). To mitigate this risk, Vauzella first introduced intraoperative wake-up testing in 1973 as a means to detect spinal cord injuries during surgery (Vauzelle et al., 1973). Although intraoperative neurophysiological monitoring (IONM) is now extensively employed in SOSCS to prevent neurological injury, its effectiveness can be influenced by surgical manipulation, anesthetic medications, blood pressure, and temperature fluctuations. Consequently, intraoperative wake-up testing remains the “gold standard” for assessing neural pathway integrity during SOSCS (Lee et al., 2006; Daniel et al., 2018).

Currently, sedation regimens for intraoperative wake-up testing during SOSCS, such as propofol alone or in combination with dexmedetomidine, are commonly utilized in clinical practice. However, these methods exhibit several drawbacks, including hemodynamic instability, poor patient coordination, and prolonged wake-up times (Ebert et al., 2000; Imani et al., 2006; Chen et al., 2018; Sahinovic et al., 2018). Achieving high-quality intraoperative wake-up responses thus remains challenging. Optimal wake-up testing requires that patients quickly emerge from anesthesia, comprehend, and respond accurately to physician instructions, yet subsequently experience amnesia regarding the wake-up event without suffering psychological trauma. This demands meticulous anesthetic management and the selection of short-acting, easily controllable sedatives capable of inducing reliable anterograde amnesia. Remimazolam, an ultra-short-acting GABAA receptor agonist, is characterized by rapid onset, fast metabolism, lack of cumulative effects during continuous infusion, and swift antagonism by flumazenil. Due to its ultra-short-acting profile and precise controllability, remimazolam may offer distinct advantages in achieving targeted anesthesia and rapid emergence (Allen and Goettel, 2025). Previous studies (Sato et al., 2020) has indicated that remimazolam facilitates rapid and high-quality emergence during craniotomy wake-up tests; however, its utility in SOSCS remains insufficiently investigated.

This study aims to assess the effectiveness and safety of remimazolam combined with propofol in scoliosis surgery, comparing it to traditional sedation approaches. Specifically, it evaluates wake-up time, wake-up quality, IONM outcomes, hemodynamic stability, cerebral state indices, and postoperative adverse reactions, to optimize anesthetic strategies.

## 2 Materials and methods

### 2.1 Study design

This blind, randomized controlled trial was approved by the Institutional Review Board of the First People’s Hospital of Yunnan Province (KHLL2023-KY136) and registered with the Chinese Clinical Trial Registry (ChiCTR2300076802). The registration occurred prior to the start of the trial and any patient enrollment undertaken. The study adhered to the Declaration of Helsinki and CONSORT guidelines (Schulz et al., 2010), and all participants (or legally authorized representatives of pediatric patients) provided written informed consent.

### 2.2 Participants

Between October 2023 and June 2025, 54 patients scheduled to undergo idiopathic SOSCS at the First People’s Hospital of Yunnan Province (Kunming, China) were recruited and randomly assigned into three groups (n = 18 per group): the propofol group (Group P), the propofol–dexmedetomidine combination group (Group D), and the propofol–remimazolam combination group (Group R). The inclusion criteria were: (I) patients scheduled for elective SOSCS; (II) American Society of Anesthesiologists (ASA) physical status I–III; (III) age 10–50 years; and (IV) ability and willingness to provide informed consent. The exclusion criteria included: (I) presence of organic central nervous system disorders or impaired consciousness; (II) severe coagulopathy; (III) severe comorbidities, such as severe pulmonary hypertension, hepatic encephalopathy, acute myocardial infarction, acute heart failure, or hepatorenal failure; (IV) psychiatric disorders or communication barriers precluding cooperation during wake-up testing; (V) known allergy to any study medication; (VI) history of substance abuse or opioid tolerance; (VII) conditions including epilepsy, cortical lesions, cranial defects, intracranial hypertension, or presence of intracranial hardware; and (VIII) any condition deemed unsuitable for study participation by the investigators. Withdrawal criteria were: (I) occurrence of massive hemorrhage or neural injury during surgery; (II) prolonged surgery duration exceeding 8 h; (III) suspicion of malignant hyperthermia; and (IV) inability to obtain baseline neurophysiological potentials within 30 min.

### 2.3 Randomization and blinding

After obtaining informed consent, participants were randomly allocated to groups using a computer-generated randomization sequence with a 1:1:1 ratio, implemented using sequentially numbered, sealed, opaque envelopes. The randomization sequence was securely stored with restricted access to ensure confidentiality. During data analysis, the groups were labeled as “Group A,” “Group B,” and “Group C” until statistical analysis was complete. Blinding was maintained for patients, surgeons, neurophysiological monitoring specialists, and outcome assessors (anesthesia assistants). However, anesthesiologists were necessarily unblinded due to pharmacological differences requiring individualized dosing adjustments for patient safety. All outcome measurements were conducted by trained assessors who remained blinded to group assignments.

### 2.4 Interventions

Standardized anesthetic protocols and monitoring were applied for all groups. Brain state index was monitored continuously using a multifunctional device (HXD20231017, Beijing Yifei Huatong Technology Development Co., Ltd., China). Assessments were performed at six time points: upon entering the operating room (T0), after anesthesia induction (T1), after exposing the spinal surgical area (T2), 20 min before intraoperative awakening (T3), immediately upon awakening (T4), and 10 min after surgery conclusion (T5). Parameters assessed included depth of sedation, analgesic effectiveness, pain levels, indices of cerebral activity, anxiety, amnesia, and incidence of delirium and agitation. The sedation index (WLi) monitored anesthetic depth, with a target range of 40–60 (Herzog et al., 2021). The analgesia index (PTi) indicated pain tolerance (Liu et al., 2017; Wu et al., 2019); higher values represented lower tolerance. The pain index (Pi) correlated with pain scales (Wu et al., 2019). The subcortical excitation index (SCEi) and cortical excitation index (CEi) measured anesthetic effects on the central nervous system (Liu, 2024).

Following topical anesthesia of the pharyngeal mucosa with 2% lidocaine, anesthetic induction was initiated. Anesthetic induction was standardized across all groups. The regimen included atropine (2303101#, Henan Runhong Pharmaceutical Co., Ltd., Xinzheng, China) at a dose of 0.01 mg/kg, dexamethasone (230302312#, Shanghai Pujin Linzhou Pharmaceutical Co., Ltd., Shanghai, China) at 2–10 mg, and etomidate (TYT23K54#, Jiangsu Hengrui Pharmaceutical Co., Ltd., Xuzhou, China) at 0.15–0.3 mg/kg. Upon achieving loss of consciousness, sufentanil (31A120212#, Yichang Humanwell Pharmaceutical Co., Ltd., Yichang, China) at 0.3–0.6 μg/kg and vecuronium (GA2305.1, Zhejiang Xianju Pharmaceutical Co., Ltd., Taizhou, China) at 0.1 mg/kg were administered. Endotracheal intubation was performed when WLi reached 60. Mechanical ventilation parameters were set to a tidal volume of 8–10 mL/kg and respiratory rate of 10–12 breaths per minute, maintaining end-tidal CO_2_ at 35–40 mmHg. Anesthetic maintenance involved standardized analgesia, utilizing continuous infusion of remifentanil (30A11181#, Yi chang Human well Pharmaceutical Co., Ltd., Yichang, China) at 0.25–0.4 μg/kg/min, targeting PTi values of 40–60. Sedation protocols varied among groups as follows: Group P received propofol (batch number 23081780, Chongqing Yaoyou Pharmaceutical Co., Ltd., Chongqing, China) at 4–10 mg/kg/h. Group D received propofol at 4 mg/kg/h combined with dexmedetomidine (batch number 23052331, Shijiazhuang No.4 Pharmaceutical Co., Ltd., Shijiazhuang, China) at 0.2–1.0 μg/kg/h. Group R received propofol at 4 mg/kg/h combined with remimazolam (batch number 90T0703, Yichang Humanwell Pharmaceutical Co., Ltd., Yichang, China) at 0.5–1.0 mg/kg/h. All sedation protocols maintained WLi between 40 and 60. Nasopharyngeal temperature was continuously monitored, and hemodynamic parameters were maintained within 20% of baseline.

Neurophysiological monitoring was performed according to established protocols (Angelliaume et al., 2023) using an intraoperative monitoring system (Protektor 32, Natus Medical Incorporated DBA Excel-tech Ltd. [XLTEK], Oakville, Canada). Somatosensory evoked potentials (SEPs) and motor evoked potentials (MEPs) were assessed at key timepoints: post-surgical exposure (t1), post-pedicle screw insertion (t2), and post-spinal correction (t3) to evaluate sensory and motor pathway integrity. SEPs were recorded by stimulating the posterior tibial nerve with spiral electrodes (30–60 mA, 0.2–0.3 m, 5 Hz) and obtaining potentials at Cz–Fz. MEPs were monitored by multi-pulse stimulation of the extensor hallucis longus or tibialis anterior muscles (300–1000 V, 0.1–0.3 m, 2 m intervals), with corresponding muscle recordings. Warning criteria included amplitude reduction >50% or latency prolongation >10%, and stimulation parameters were adjusted accordingly.

Wake-up testing protocols included preoperative patient education regarding intraoperative awakening and cooperation with commands. Vecuronium administration was discontinued 60 min prior to awakening to minimize neuromuscular blockade. In accordance with established methods (Sato et al., 2020; Sato and Nishiwaki, 2022; Viderman et al., 2023), wake-up preparation involved reducing remifentanil to 0.05 μg/kg/min in all groups to maintain analgesia. Group-specific interventions were as follows: Group P discontinued propofol; Group D discontinued propofol and dexmedetomidine; Group R discontinued propofol and remimazolam, with administration of flumazenil (TFM23105#, Jiangsu Hengrui Pharmaceutical Co., Ltd., Xuzhou, China) at 0.01 mg/kg intravenously. Baseline wake-up WLi values, as determined by pilot data, were 79.1 (4.0) for Group P, 79.6 (4.4) for Group D, and 80.3 (7.1) for Group R. Wake-up assessment began when WLi approached 70, with evaluations conducted every 30 s by calling the patient’s name and observing responses (eye opening, finger/toe movement). The duration and quality of the wake-up process were systematically documented (Quan et al., 2016).

The postoperative analgesia protocol included surgical wound infiltration with 0.25% ropivacaine, supplemented by patient-controlled analgesia (PCA) delivering sufentanil (1.5–2 μg/kg). Anesthesia assistants recorded the satisfaction levels of surgeons and patients using a 0–10 Numerical Rating Scale (NRS), categorized as follows: 9–10 (very satisfied), 7–8 (satisfied), 4–6 (neutral), 3 (dissatisfied), and 0–2 (very dissatisfied) (Cortellazzo Wiel et al., 2022). Pain intensity and comfort levels were assessed using the Visual Analog Scale (VAS) (Karcioglu et al., 2018) and the Behavioral Comfort Scale (BCS) (Huang et al., 2023) at 2, 6, 12, and 24 h post-surgery. Additional data collected included the duration of surgery, pharmacological use, and occurrence of adverse events such as postoperative nausea and vomiting, sleep disturbances, dizziness, and headache.

### 2.5 Outcomes

#### 2.5.1 Primary outcomes

The primary outcomes included wake-up time (defined as the interval from the start of testing to patient arousal) and wake-up quality. Wake-up quality was assessed using validated criteria (Chen et al., 2018): (I) Excellent (3 points), calm and responsive with appropriate limb movement; (II) Good (2 points), sudden awakening with involuntary limb movement not affecting surgical equipment; (III) Poor (1 point), sudden awakening with violent movements requiring restraint to protect instruments.

#### 2.5.2 Secondary outcomes

Secondary outcomes included neurophysiological monitoring parameters, cerebral state indices, hemodynamic variables, and satisfaction scores. Additional measures included duration of anesthesia and surgery, drug consumption, and incidence of adverse events.

### 2.6 Sample size

Sample size calculation was performed using PASS 15.0 software (NCSS LLC, Kaysville, United States). Preliminary pilot study data indicated mean wake-up times of 12.40 min for Group P, 21.60 min for Group D, and 5.00 min for Group R, with corresponding standard deviations of 1.51 min, 3.50 min, and 1.22 min, respectively. A power analysis (α = 0.05, two-sided; power = 0.90) suggested 12 participants per group. To account for potential attrition, final enrollment was increased to 18 participants per group.

### 2.7 Statistical analysis

Statistical analyses were conducted using SPSS version 27.0 (IBM Corporation, Armonk, NY, United States). Categorical variables were expressed as frequencies and percentages and analyzed using chi-square or Kruskal–Wallis tests. Continuous variables with a normal distribution were reported as means ± standard deviations (SD) and analyzed using ANOVA with Bonferroni correction for multiple comparisons. Continuous and ordinal variables without a normal distribution were presented as medians with interquartile ranges (IQR) and analyzed using the Kruskal–Wallis test. Statistical significance was set at *p* < 0.05.

## 3 Result

**FIGURE 1:**
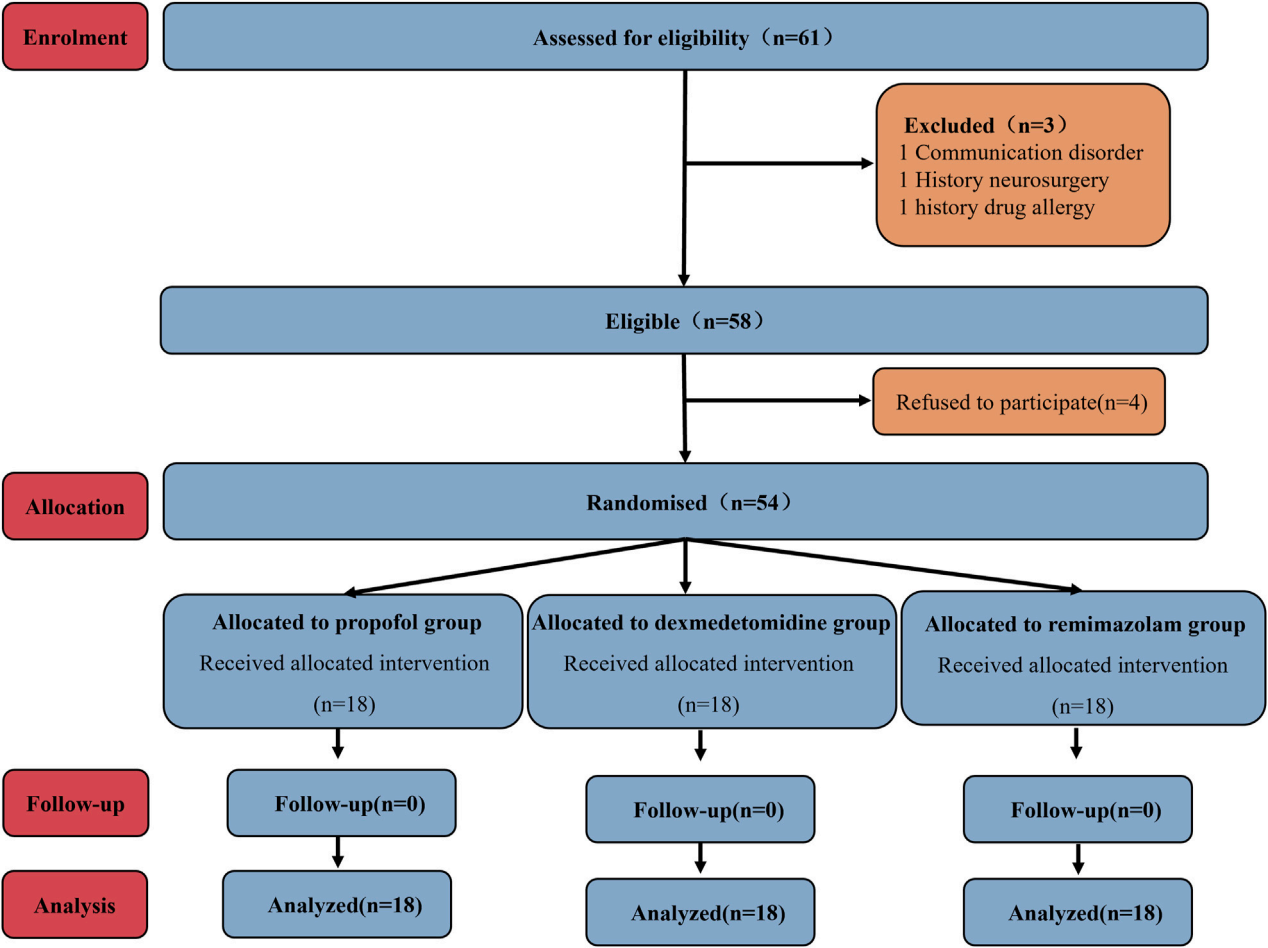
CONSORT study flow chart.

A total of 61 patients were initially recruited; 4 patients declined participation. The remaining 57 patients were randomly assigned to Groups P, D, and R. Subsequently, exclusions occurred as follows: one patient from Group P due to communication barriers, one patient from Group D due to a documented history of drug allergy, and one patient from Group R due to prior cranial surgery. Ultimately, each group comprised 18 participants for analysis (Figure 1). As shown in Table 1, no statistically significant differences existed among the three groups regarding baseline demographic and clinical characteristics (age, gender, BMI, ASA classification, anesthesia induction time, and pre-awakening medication maintenance duration).

**TABLE 1:** Patient characteristics at baseline.

Variable	Group P (*N* = 18)	Group D (*N* = 18)	Group R *(N* = 18)	*p-value*
Age (year), median [IQR]	14.0 [12.0-16.3]	16.0 [14.0-29.8]	19.5 [13.0-34.0]	0.16^a^
Gender				0.37^b^
Male, n (%)	4 (22.2%)	8 (44.4%)	6 (33.3%)	
Female, n (%)	14 (77.8%)	10 (55.6%)	12 (66.7%)	
BMI (kg/m^2^), mean ± SD	19.4 ± 3.1	18.5 ± 4.1	19.2 ± 3.6	0.73^c^
ASA, n (%)				0.73^b^
Ⅱ	14 (77.8%)	13 (72.2%)	15 (83.3%)	
Ⅲ	4 (22.2%)	5 (27.8%)	3 (16.7%)	
Pre-awakening medication duration (min), mean ± SD	184.2 ± 57.6	228.1 ± 56.9	183.3 ± 69.9	0.05^c^

IQR, interquartile range; SD, standard deviation; ASA, american society of anesthesiologists; BMI, body mass index.

^a^ Kruskal–Wallis test.

^b^ Chi-square test.

^c^ ANOVA.

### 3.1 Primary outcomes

Regarding primary outcomes (Table 2), the wake-up times differed significantly among groups: Group P had a mean of 10.58 ± 1.60 min (95% CI, 9.78–11.38), Group D 16.14 ± 2.64 min (95% CI, 14.83–17.45), and Group R 6.69 ± 3.85 min (95% CI, 4.77–8.61). Post-hoc analysis indicated that Group R had significantly shorter wake-up times compared to Group P (mean difference 3.9 min, 95% CI: 1.6–6.2, Bonferroni-corrected *p* = 0.0005) and Group D (mean difference 9.4 min, 95% CI: 7.1–11.8, Bonferroni-corrected *p* = 0.0001). Additionally, excellent wake-up quality was observed in 9 (50%) patients in Group P, 18 (100%) in Group D, and 17 (94.4%) in Group R (*p* = 0.0002). The mean operative durations also differed significantly among groups: Group P, 264.4 ± 60.4 min (95% CI, 234.4–294.5); Group D, 310.9 ± 57.9 min (95% CI, 282.1–339.7); and Group R, 256.3 ± 74.4 min (95% CI, 219.3–293.3) (*p* = 0.03). Post-hoc analysis revealed that Group R had significantly shorter operative durations compared to Group D (*p* = 0.04), while other pairwise comparisons were not statistically significant (*p* > 0.05).

**TABLE 2:** Evaluation metrics for wake-up tests.

Variable	Group P (*N* = 18)	Group D (*N* = 18)	Group R *(N* = 18)	*p-value*
Wake-up time (min), mean ± SD	10.6 ± 1.6	16.1 ± 2.6	6.7 ± 3.8	0.0001^a^
*p* value compared with group P *p* value compared with group D *p* value compared with group R	NA 0.0001 0.0005	0.0001 NA 0.0001	0.0005 0.0001 NA	
Wake-up quality, n (%) 2 points 3 points	9 (50%) 9 (50%)	0 (0%) 18 (100%)	1 (5.6%) 17 (94.4%)	0.0002^b^
Surgical time (min), mean ± SD	264.4 ± 60.4	310.9 ± 57.9	256.3 ± 74.4	0.03^a^
*p* value compared with group P *p* value compared with group D *p* value compared with group R	NA 0.11 0.99	0.11 NA 0.04	0.99 0.04 NA	
Induction to extubation time (min), mean ± SD *p* value compared with group P *p* value compared with group D *p* value compared with group R	306.6 ± 66.8 NA 0.05 1.00	363.4 ± 63.9 0.05 NA 0.02	300.4 ± 77.5 1.00 0.02 NA	0.02^a^
Surgeon Satisfaction (point), median [IQR] *p* value compared with group P *p* value compared with group D *p* value compared with group R	5 [3.8-7.0] NA 0.29 0.005	4.0 [3.0-5.0] 0.29 NA 0.0001	7.5 [6.0-8.3] 0.005 0.0001 NA	0.0001^c^
Patient satisfaction (point) median [IQR] *p* value compared with group P *p* value compared with group D *p* value compared with group R	5.0 [4.0-6.0] NA 0.0004^d^ 0.0001^d^	7.0 [6.8-8.0] 0.0004^d^ NA 0.99^d^	8.0 [6.8-8.3] 0.0001^d^ 0.99^d^ NA	0.0001^c^

IQR, interquartile range; SD, standard deviation; NA, not applicable.

^a^ Bonferroni’s multiple comparisons test.

^b^ Chi-square test.

^c^ Kruskal–Wallis test.

^d^ Dunn’s multiple comparisons test.

### 3.2 Secondary outcomes

Surgeon satisfaction scores (median [IQR]) were 5.0 (3.8–7.0) for Group P, 4.0 (3.0–5.0) for Group D, and 7.5 (6.0–8.3) for Group R, indicating significant differences (*p* = 0.0001). Post-hoc analysis confirmed that Group R achieved significantly higher surgeon satisfaction scores compared to Group P (*p* = 0.005) and Group D (*p* = 0.0001). Patient satisfaction scores (median [IQR]) were 5.0 (4.0–6.0) for Group P, 7.0 (6.8–8.0) for Group D, and 8.0 (6.8–8.3) for Group R, also demonstrating significant differences (*p* = 0.0001). Post-hoc analysis indicated significantly higher patient satisfaction scores in Group R compared to Group P (*p* = 0.0001), while no significant difference was observed compared to Group D (*p* = 0.99) (Table 2).

**FIGURE 2:**
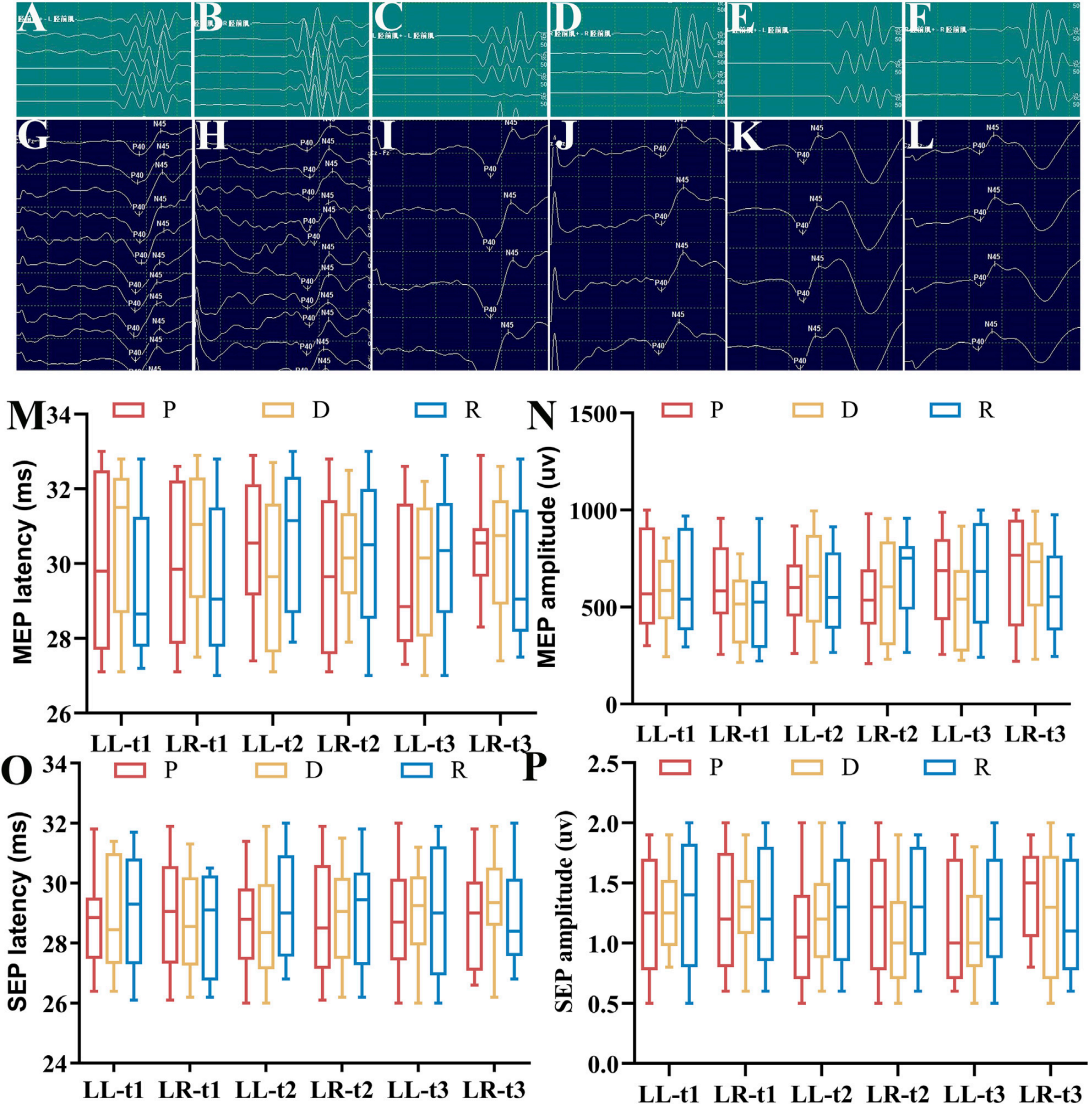
Lower Limb SEP and MEP Latency and Amplitude. **(A)** Right lower limb MEP waveform (Group P); **(B)** Left lower limb MEP waveform (Group P); **(C)** Right lower limb MEP waveform (Group D); **(D)** Left lower limb MEP waveform (Group D); **(E)** Right lower limb MEP waveform (Group R); **(F)** Left lower limb MEP waveform (Group R); **(G)** Right lower limb SEP waveform (Group P); **(H)** Left lower limb SEP waveform (Group P); **(I)** Right lower limb SEP waveform (Group D); **(J)** Left lower limb SEP waveform (Group D); **(K)** Right lower limb SEP waveform (Group R); **(L)** Left lower limb SEP waveform (Group R); **(M)** Latency statistics for lower limb MEP at timepoints t1, t2, t3 [M (P25-P75)]; **(N)** Amplitude statistics for lower limb MEP at timepoints t1, t2, t3 [M (P25-P75)]; **(O)** Latency statistics for lower limb SEP at timepoints t1, t2, t3 [M (P25-P75)]; **(P)** Amplitude statistics for lower limb SEP at timepoints t1, t2, t3 [M (P25-P75)]. All data sets were analyzed using the Kruskal–Wallis test. LL, left lower limb; LR, right lower limb; t1, after exposure of the spinal surgical area; t2, after pedicle screw insertion; t3, after spinal alignment.

**FIGURE 3:**
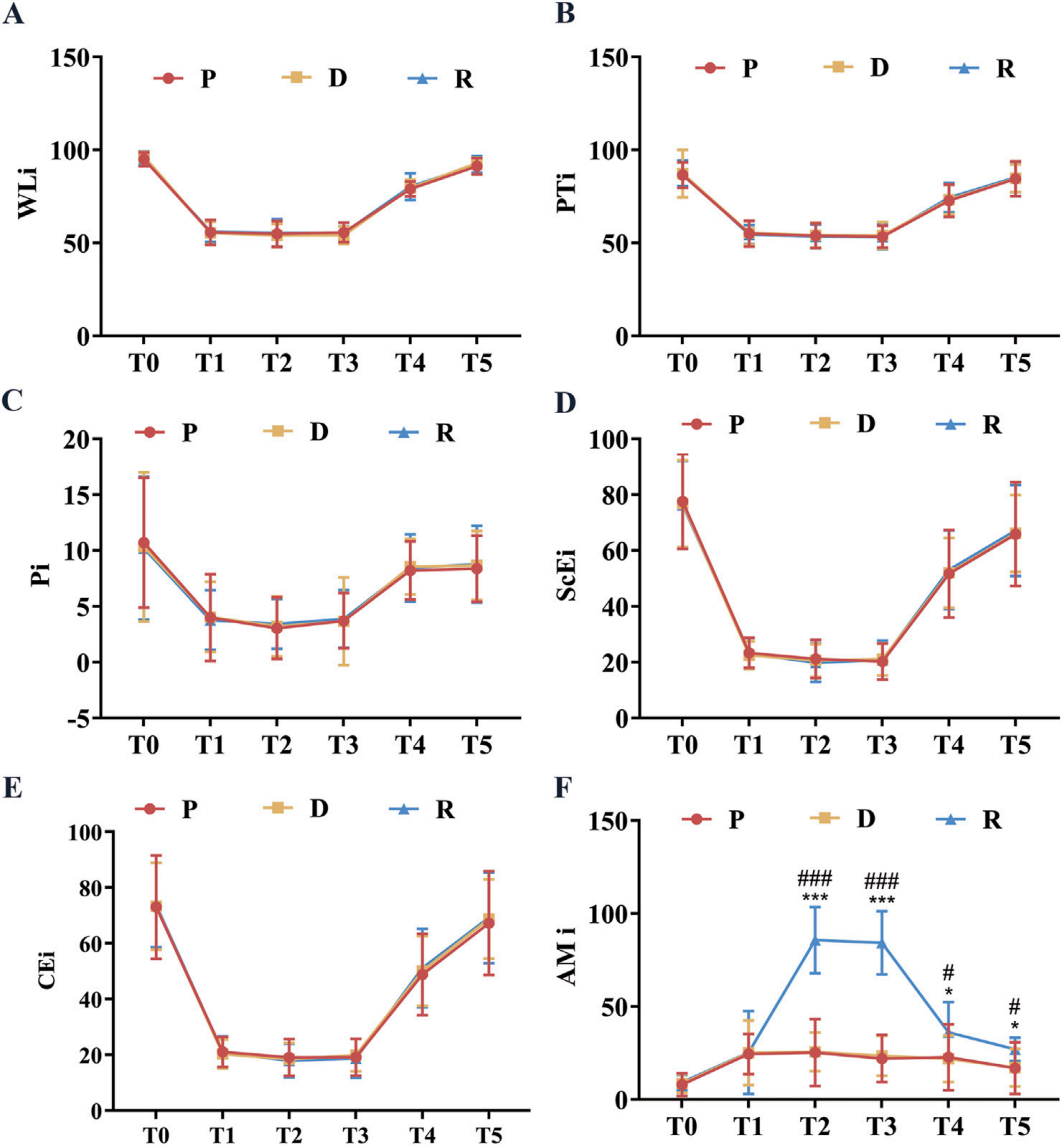
Perioperative Brain State Indexes. **(A)** WLi at various perioperative time points; **(B)** PTi at various perioperative timepoints; **(C)** Pi at various perioperative time points; **(D)** SCEi at various perioperative time points; **(E)** CEi at various perioperative time points; **(F)** AMi at various perioperative timepoints. All data are expressed as mean ± standard deviation (SD), analyzed by ANOVA, followed by Bonferroni’s *post hoc* multiple comparisons test. Compared to Group P, * represents *p* < 0.05, *** represents *p* < 0.001; compared to Group P, # represents *p* < 0.05, ### represents *p* < 0.001. Upon entering the operating room (T0), after anesthesia induction (T1), after exposing the spinal surgical area (T2), 20 min before intraoperative awakening (T3), immediately upon awakening (T4), and 10 min after surgery conclusion (T5).

**FIGURE 4:**
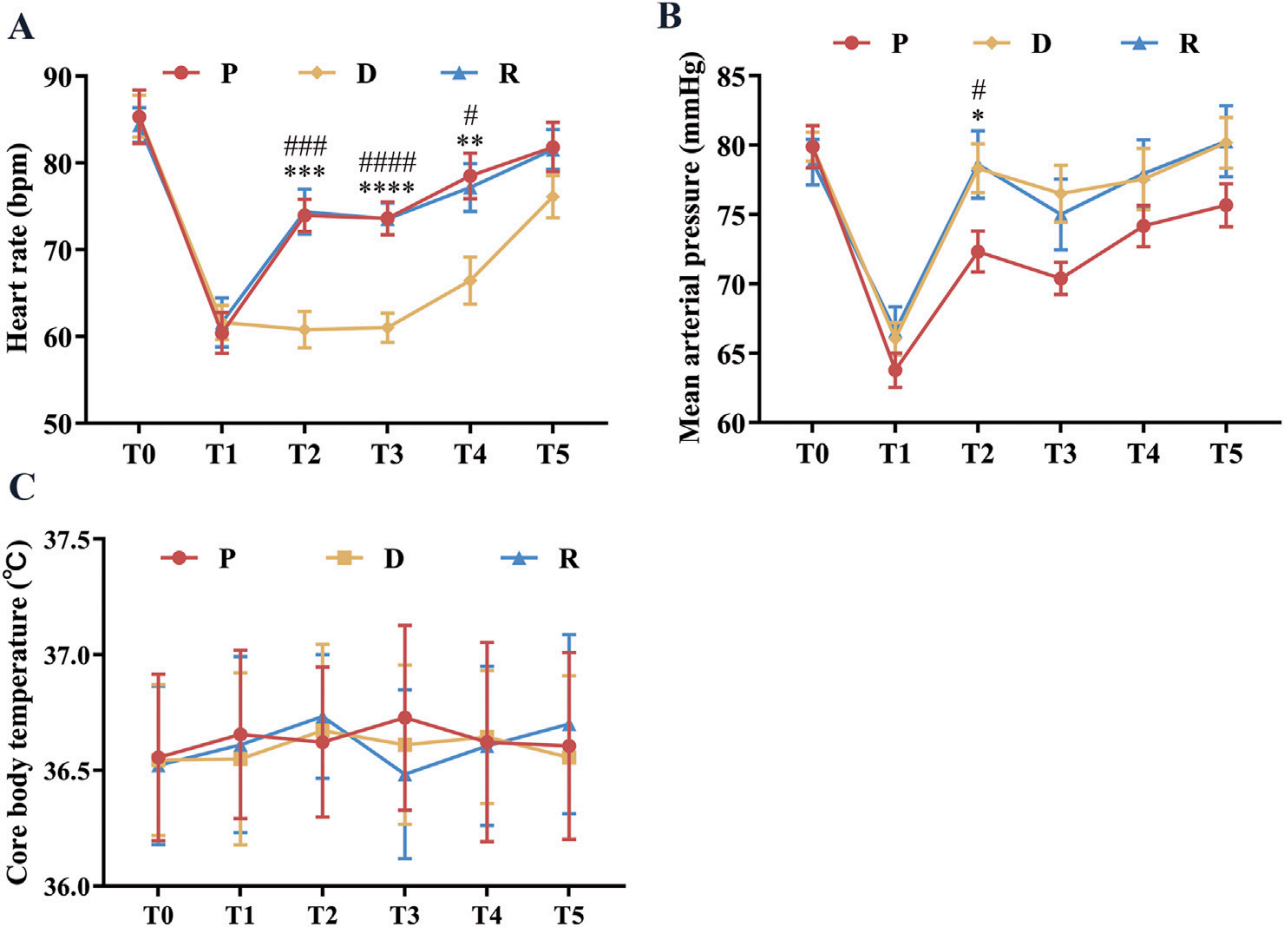
Perioperative Hemodynamics and Core Temperature. **(A)** Represents the heart rate at different perioperative time points. **(B)** Represents the mean arterial pressure at different perioperative time points. **(C)** Represents the mean Core body Temperature at different perioperative time points. All data are expressed as mean ± standard deviation (SD), analyzed by ANOVA, followed by Bonferroni’s multiple comparisons test for *post hoc* analysis. Compared to group P, * represents *p* < 0.05, ** represents *p* < 0.01; *** represents *p* < 0.001, **** represents *p* < 0.0001, compared to group D, ### represents *p* < 0.001; #### represents *p* < 0.0001. Upon entering the operating room (T0), after anesthesia induction (T1), after exposing the spinal surgical area (T2), 20 min before intraoperative awakening (T3), immediately upon awakening (T4), and 10 min after surgery conclusion (T5).

The spinal neurophysiological monitoring results (Figures 2A–P) showed no statistically significant differences in the latencies and amplitudes of lower extremity SEPs and MEPs at timepoints t1, t2, and t3 among Groups P, D, and R (*p* > 0.05). Cerebral state monitoring revealed significantly higher amnesia indices (AMi) in Group R compared to Groups P and D at timepoints T2, T3, T4, and T5 (*p* < 0.001, Figure 3F). No statistically significant differences were observed among the three groups for other cerebral state parameters, including WLi, PTi, Pi, SCEi, CEi, and CFi, at timepoints T0–T5 (*p* > 0.05, Figures 3A–E). Hemodynamic monitoring indicated that Group D had significantly lower heart rates at timepoints T2, T3, and T4 compared to Groups P and R (*p* < 0.05, Figure 4A). At timepoint T2, Groups D and R exhibited significantly higher mean arterial pressure compared to Group P (*p* < 0.05, Figure 4B). Core body temperatures remained stable and consistent across all three groups during the perioperative period (T0–T5) (*p* > 0.05, Figure 4C).

Analysis of additional outcome measures indicated no statistically significant differences among Groups P, D, and R regarding the consumption of anesthetic drugs (sufentanil, etomidate, vecuronium, and remifentanil; *p* > 0.05, Table 3). Moreover, postoperative pain assessment (VAS scores) and comfort evaluation (BCS scores) at 2, 6, 12, and 24 h postoperatively showed no significant differences among the three groups (*p* > 0.05, Table 4). Additionally, the incidence of adverse events within 24 h postoperatively did not differ significantly among Groups P, D, and R (*p* > 0.05).

**TABLE 3:** Comparison of drug consumption.

Variable	Group P (*N* = 18)	Group D (*N* = 18)	Group R *(N* = 18)	*p-value*
Sufentanil induction dose (ug/kg), median [IQR]	0.63 [0.53-0.78]	0.62 [0.55-0.73]	0.70 [0.59-0.97]	0.25^a^
Vecuronium induction dose (mg/kg), median [IQR]	0.12 [0.10-0.13]	0.09 [0.08-0.13]	0.12 [0.10-0.14]	0.12^a^
Etomidate induction dose (mg/kg), median [IQR]	0.38 [0.30-0.43]	0.35 [0.29-0.43]	0.38 [0.36-0.47]	0.35^a^
Remifentanil maintenance dose (ug/kg/min), mean ± SD	0.25 ± 0.03	0.22 ± 0.02	0.23 ± 0.05	0.11^a^
Propofol maintenance dose (mg/kg/h), mean ± SD	5.17 ± 0.90	4.090 ± 0.77	4.14 ± 0.82	0.0002^a^
*p* value compared with group P *p* value compared with group D *p* value compared with group R	NA 0.0006^b^ 0.002^b^	0.0006^b^ NA 1.0^b^	0.002^b^ 1.0^b^ NA	

IQR, interquartile range; SD , standard deviation; NA = not applicable.

^a^ Kruskal–Wallis test.

^b^ Dunn’s multiple comparisons test.

**TABLE 4:** Postoperative outcome and adverse reactions.

Variable	Group P (*N* = 18)	Group D (*N* = 18)	Group R *(N* = 18)	*p-value*
Hospitalization time (day), median [IQR]	12.0 [9.8-14.0]	13.0 [9.0-15.0]	11 [8.8-13.0]	0.46^a^
Postoperative VAS score, median [IQR]				
2 h	4.0 [2.8-6.0]	5.0 [3.0-6.0]	4.0 [3.0-6.0]	0.89^a^
6 h	5.0 [4.8-6.3]	5.0 [4.8-6.3]	5.0 [4.0-6.0]	0.86^a^
12 h	5.5 [5.0-7.0]	6.0 [5.0-7.0]	5.5 [4.8-6.3]	0.67^a^
24 h	3.5 [3.0-4.3]	3.0 [3.0-4.0]	4.0 [3.0-4.3]	0.36^a^
Postoperative BCS score, median [IQR]				
2 h	0 [0-1]	0 [0-1]	0 [0-1]	0.73^a^
6 h	0 [0-0]	0 [0-0]	0 [0-0]	0.77^a^
12 h	0 [0-0]	0 [0-0]	0 [0-0.3]	0.89^a^
24 h	1 [1-2]	1 [1-2]	0.8 [1-2]	0.36^a^
Postoperative adverse reactions, n (%)				
PONV	5 (27.7%)	5 (27.7%)	7 (38.9%)	1.00^a^
Insomnia	3 (16.7%)	2 (11.1%)	3 (16.7%)	1.00^a^
Dizziness、headache	0 (0%)	2 (11.1%)	0 (0%)	0.32^a^

IQR, interquartile range; VAS, visual analogue scale; BCS, bruggemann comfort scale.

^a^ Kruskal–Wallis test.

^b^ Dunn’s multiple comparisons test.

## 4 Discussion

During SOSCS, various perioperative factors can result in iatrogenic spinal cord injury. These factors include nerve traction or compression during spinal decompression procedures, pedicle screw instrumentation, osteotomy, and inadequate spinal cord perfusion due to intraoperative hemodynamic instability (Wilson-Holden et al., 1999; Delank et al., 2005; Rahyussalim et al., 2016). Previous studies (Hamilton et al., 2011) reported an incidence of neurological complications following adult spinal SOSCS of 1.84%, compared to 0.99% in pediatric populations. Additional research demonstrated that spinal deformity correction performed without neurophysiological monitoring significantly increased the risk of neurological injury, potentially causing motor and sensory dysfunction or irreversible conditions such as paraplegia (Delank et al., 2005). Therefore, the implementation of wake-up testing and comprehensive spinal neurophysiological monitoring during SOSCS is crucial. These interventions enable early detection of potential neurological compromise and allow timely therapeutic interventions, thereby preventing catastrophic outcomes.

Intraoperative wake-up testing and IONM are currently the most widely used methods for neural monitoring during surgery. Previous studies (Tamkus et al., 2018) reported that IONM is susceptible to interference from anesthetic agents, body temperature fluctuations, and blood pressure variations. These factors can cause false-positive or false-negative results, potentially compromising the accurate detection of iatrogenic spinal cord or peripheral nerve injuries. Consequently, high-quality wake-up testing remains a fundamental approach for ensuring spinal cord integrity (Acharya et al., 2017; Elnady et al., 2017). The wake-up protocol requires patients to remain calm, maintain stable vital signs, and respond appropriately to commands. However, its clinical application frequently encounters challenges, such as delayed emergence from excessive anesthetic depth, pain or agitation resulting from insufficient anesthesia, and potentially severe incidents like displacement of surgical instruments or endotracheal tubes (Schulze et al., 2015). Such difficulties make it challenging to achieve optimal wake-up testing (Paunikar et al., 2023).

In recent clinical practice, sedative agents such as propofol and dexmedetomidine have increasingly been utilized during wake-up testing in spinal and neurosurgical procedures (Imani et al., 2006; Chen et al., 2018). However, propofol use is frequently associated with significant hemodynamic instability and poor patient cooperation. Although dexmedetomidine provides enhanced sedation and improved wake-up quality compared to propofol, its clinical use is limited due to prolonged emergence times and a risk of bradycardia (Ebert et al., 2000; Sahinovic et al., 2018). Therefore, sedation-based wake-up protocols relying on propofol or dexmedetomidine often do not achieve the standards for high-quality emergence. Remimazolam, an ultra-short-acting GABA receptor agonist, is characterized by rapid onset and clearance, allowing swift reversal with flumazenil and exhibiting significant anterograde amnesia effects. These features position remimazolam as an ideal sedative agent (Noor et al., 2021; Hu et al., 2022). Consequently, the pharmacological properties of remimazolam suggest significant potential benefits for use in wake-up testing protocols.

Wake-up time and wake-up quality are critical parameters for evaluating wake-up testing. In this study, Group R demonstrated a mean (SD) wake-up time of 6.69 (3.85) minutes, significantly shorter than Group P (10.58 [1.60] minutes) and Group D (16.14 [2.64] minutes). These results align with previous research examining wake-up times associated with propofol and dexmedetomidine during similar procedures (Canbay et al., 2015; Yang et al., 2022). Pharmacokinetic properties of sedative agents likely account for the differences in wake-up times. Specifically, the elimination half-lives (t_1/2_) of these agents differ: approximately 30–60 min for propofol, around 2 h for dexmedetomidine, and approximately 1 h for remimazolam. The considerably longer elimination half-life of dexmedetomidine compared to propofol and remimazolam likely explains the significantly prolonged wake-up times observed in Group D relative to Groups P and R. Although propofol and remimazolam share similar elimination half-lives, the sedative effect of remimazolam can be rapidly reversed by administering flumazenil, unlike propofol and dexmedetomidine, which lack specific antagonists. This unique pharmacological characteristic provides remimazolam with a notable advantage over propofol and dexmedetomidine, facilitating quicker emergence from sedation. This likely accounts for the superior wake-up performance observed in Group R. In terms of wake-up quality, Groups R and D demonstrated comparable outcomes, with both outperforming Group P. The shorter wake-up time in Group R also contributed to reduced operative duration, with a mean (SD) surgical time of 256.3 (74.4) minutes compared to 264.4 (60.4) minutes for Group P and 310.9 (57.9) minutes for Group D.

Wake-up time, wake-up quality, and AMi are key determinants of satisfaction ratings. In this study, surgeon satisfaction scores were higher in Group R compared to Groups P and D, likely due to faster wake-up times and superior wake-up quality in Group R.41 The inherent anterograde amnestic properties of remimazolam, absent in propofol and dexmedetomidine, corresponded to significantly higher AMi observed in Group R during wake-up episodes compared to Groups P and D. This pharmacological characteristic likely enhanced patient satisfaction scores in Group R. Considering wake-up time, wake-up quality, and satisfaction ratings, the combination of remimazolam and propofol appears optimal as a sedation regimen for wake-up testing in SOSCS.

Patients with scoliosis frequently present with cardiopulmonary dysfunction, including pulmonary hypertension and cardiac pathology. Administration of anesthetic agents, surgical blood loss, and nociceptive stimulation during emergence can cause hemodynamic instability, potentially compromising intraoperative safety (Zhou et al., 2024). Therefore, maintaining hemodynamic stability is critical for successfully navigating patients through the “Sleep-Awake-Sleep” phases of wake-up testing. This study demonstrated stable heart rate and mean arterial pressure in all three groups throughout the perioperative period, with minimal fluctuations. Although Group D exhibited lower heart rates compared to Groups P and R at timepoints T2, T3, and T4, the corresponding mean arterial pressures remained within acceptable ranges without significant variability, thus preserving relative hemodynamic stability. These findings suggest that remimazolam combined with propofol provides superior hemodynamic stability during spinal correction procedures, aligning with observations by Wu et al. (2025).

Under controlled conditions ensuring comparable anesthetic depth, blood pressure, and core body temperature—parameters known to affect monitoring accuracy—spinal neurophysiological monitoring revealed no statistically significant differences among Groups R (remimazolam at 0.5–1.0 mg/kg/h with propofol), P, and D in the latencies and amplitudes of lower extremity SEPs and MEPs at timepoints t1, t2, and t3. Consistent with previous case reports, remimazolam administered at 0.5–1.5 mg/kg/h combined with remifentanil maintenance anesthesia did not result in significant alterations in SEP and MEP parameters (Kondo et al., 2020). Postoperative follow-up evaluations showed no statistically significant differences among Groups P, D, and R regarding VAS pain scores, BCS ratings, or adverse event incidence. These findings imply that the combination of remimazolam and propofol does not negatively impact postoperative pain management, patient comfort, or complication rates compared to propofol monotherapy or propofol-dexmedetomidine combinations.

In conclusion, this study demonstrates that the remimazolam-propofol combination for wake-up testing in SOSCS offers significant advantages over traditional propofol monotherapy or propofol-dexmedetomidine combinations. These advantages include shorter wake-up times, better awakening quality, improved satisfaction ratings, and enhanced controllability, all while maintaining comparable safety profiles concerning spinal neurophysiological monitoring, hemodynamic stability, postoperative pain management, and adverse event rates. Based on these findings, the combination of remimazolam and propofol constitutes an optimal sedation regimen for wake-up testing during scoliosis correction procedures.

Nonetheless, the current study has several limitations. **First**, due to intrinsic pharmacological differences among the sedatives investigated and adherence to ethical principles, we prioritized perioperative patient safety by not blinding anesthesiologists managing the patients to group allocations. This lack of blinding may have introduced bias in the titration of sedative infusions, potentially affecting secondary outcomes such as sedative consumption and hemodynamic parameters. To mitigate this bias, independent assessors blinded to group assignments collected and evaluated all outcome measures. **Second**, given the complexity and inherent risks associated with scoliosis corrective surgery, we did not conduct a trial using remimazolam as the sole sedative for the intraoperative wake-up test. Future research should evaluate remimazolam independently, potentially in lower-risk surgical populations, to thoroughly assess its standalone effects during the wake-up test. **Additionally**, the low prevalence and complex phenotypic expression of severe scoliosis restricted participant recruitment, reducing the final sample size. This limited sample size may have compromised statistical power, increasing the risk of a Type II error. Future large-scale, multicenter studies are needed to validate these preliminary findings and enhance their generalizability across diverse patient populations.

## 5 Conclusion

The use of remimazolam in combination with propofol for wake-up testing in patients undergoing SOSCS offers several advantages, including shorter wake-up times, enhanced awakening quality, robust controllability, high patient satisfaction, and induction of anterograde amnesia. Importantly, this combination does not significantly affect the latency and amplitude of SEP and MEP during neurophysiological monitoring. Administering remimazolam at doses of 0.5–1.0 mg/kg/h with propofol is both safe and effective for anesthesia management in this surgical context.

## Data Availability

The original contributions presented in the study are included in the article/supplementary material, further inquiries can be directed to the corresponding authors.
